# A Low-Power Chopper-Stabilized Readout Interface ASIC for High-Resolution TMR Magnetometers

**DOI:** 10.3390/mi17091104

**Published:** 2026-09-21

**Authors:** Wanting Rong, Dechao Sun, Wenbo Zhang, Hao Ye

**Affiliations:** 1 School of Intelligent Manufacturing, Huzhou College, Huzhou 313000, China; rongwanting1@163.com; 2 College of Artificial Intelligence, Ningbo University of Finance and Economics, Ningbo 315175, China; 3 School of Astronautics, Harbin Institute of Technology, Harbin 150001, China; zwb0311@hit.edu.cn; 4 College of Electrical and Electronic Engineering, Wenzhou University, Wenzhou 325035, China; yehao@wzu.edu.cn

**Keywords:** tunnel magnetoresistance (TMR) sensor, readout interface ASIC, programmable instrumentation amplifier (PGIA), chopper stabilization

## Abstract

Tunnel magnetoresistance (TMR) sensors have attracted considerable attention in high-resolution magnetic-field measurement owing to their high sensitivity, low power consumption, and excellent temperature stability. However, the weak differential output of TMR Wheatstone bridges is highly susceptible to DC offset and low-frequency flicker noise, which significantly limits the overall sensing performance. To address these issues, this paper presents a low-power readout interface ASIC based on a chopper-stabilized programmable instrumentation amplifier (PGIA) for TMR magnetic sensors. The proposed PGIA provides eight programmable gain settings from 1 V/V to 128 V/V. A transconductance equalization technique is introduced to maintain nearly constant input transconductance over the entire rail-to-rail common-mode input range, thereby improving gain stability and reducing input-referred noise. In addition, a dynamic slew-rate enhancement circuit is employed to improve transient response without increasing static power consumption, while a digitally assisted offset calibration circuit effectively suppresses input offset and enhances measurement accuracy. The proposed interface ASIC was fabricated using a standard 0.18 μm CMOS process and experimentally evaluated in a compact TMR magnetometer prototype. Measurement results demonstrate a full-scale nonlinearity of 0.1% FS over a ±100 μT magnetic-field range, a magnetic noise density of 0.13 nT/√Hz at 1 Hz, and a combined power consumption of 10 mW for the readout ASIC and TMR sensing bridge, with the prototype powered from a 5 V external supply and the ASIC core operating from a regulated 3.3 V rail. Compared with representative reported magnetometers, the proposed system achieves an excellent trade-off among power consumption, linearity, and magnetic-field resolution, making it well suited for portable and high-precision magnetic sensing applications.

## 1. Introduction

The rapid development of intelligent sensing, data-driven decision-making, and advanced computational methods has made reliable physical-signal acquisition an important enabling technology across diverse engineering fields. Industry 4.0-driven digital transformation has been investigated to improve the resilience and visibility of aerospace supply chains [1], while life-cycle and techno-economic assessment methods have been employed to evaluate sustainable radioactive-waste treatment and immobilization pathways [2]. The integration of digital twins and artificial intelligence has also provided new opportunities for predictive maintenance and health management in microelectromechanical systems [3]. In earth science, quantitative mapping of the global tourmaline research landscape has revealed emerging directions in mineral exploration and petrogenetic analysis [4]. Model-assisted optimization and intelligent algorithms have further been applied to balance surface quality and ablation depth in precision laser manufacturing [5], whereas deep-learning-based onboard sensing has enabled reliable detection of rail corrugation [6]. In the energy sector, combined experimental, molecular-simulation, and computational-fluid-dynamics methods have advanced the understanding of flocculation, deposition, and transport of heavy components in crude-oil storage tanks and non-Newtonian oil-wax pipelines [7,8,9]. In addition, biomimetic nanoassemblies have enabled neurostimulation-based investigation of bacterial metabolic processes, demonstrating the growing importance of sensitive and controllable bioanalytical platforms [10]. Sensor-based PUFs have increasingly attracted significant research interest in recent years [11,12,13,14,15,16,17,18,19]. Although these studies address different application scenarios, they collectively highlight the increasing demand for accurate, low-noise, robust, and energy-efficient acquisition of weak physical signals. Within this broader technological context, TMR magnetic sensors are particularly attractive because of their high sensitivity, wide dynamic range, low power consumption, and compatibility with compact electronic systems. However, fully exploiting these advantages requires a dedicated readout interface capable of suppressing bridge offset and low-frequency noise while maintaining stable gain and rail-to-rail operation.

**Figure 1:**
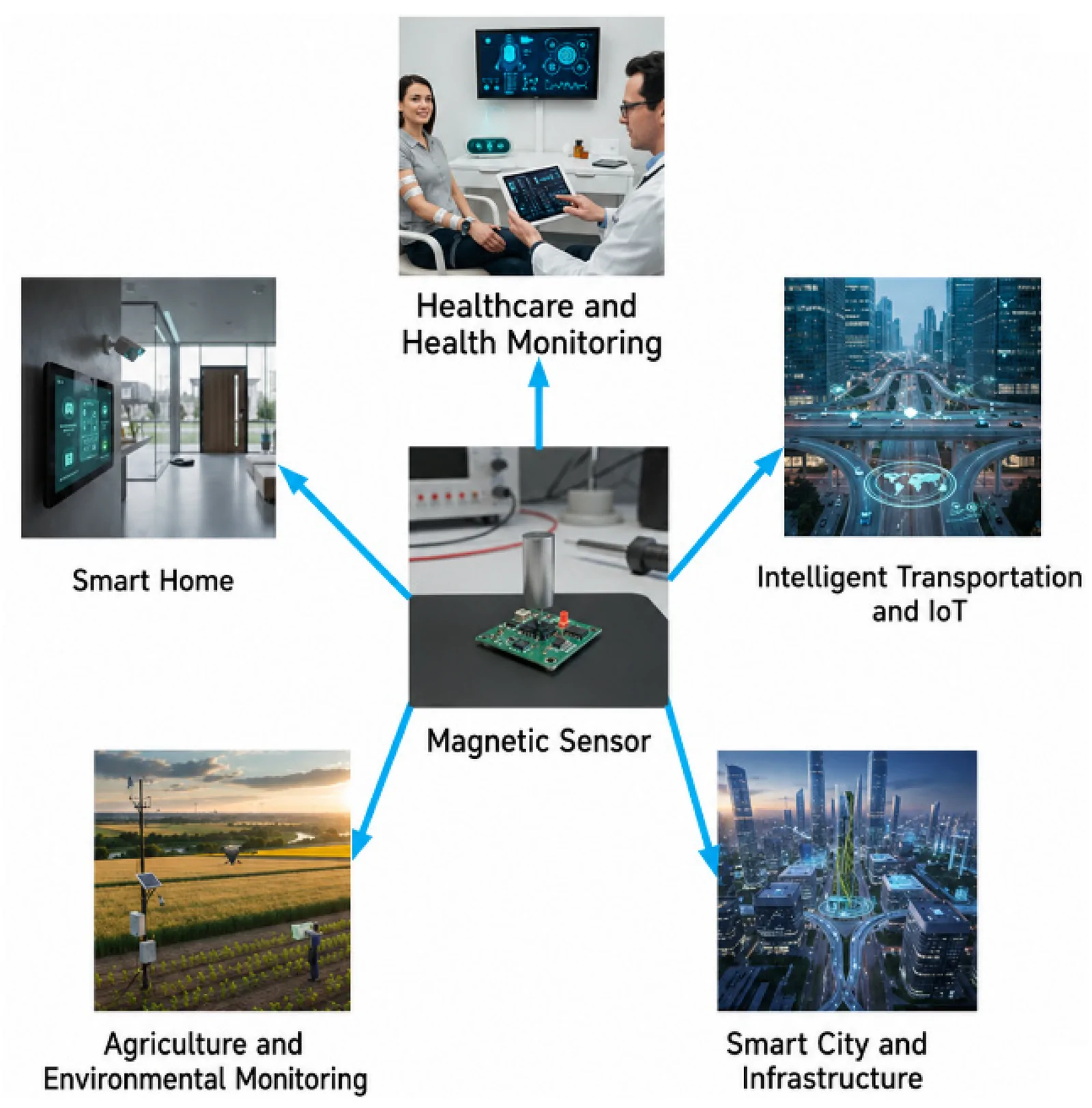
Representative applications of magnetic sensors.

Tunnel magnetoresistance (TMR) sensors have emerged as one of the most promising magnetic sensing technologies owing to their high sensitivity, wide dynamic range, excellent temperature stability, and CMOS compatibility. Compared with Hall-effect and anisotropic magnetoresistance (AMR) sensors, TMR sensors provide significantly higher sensitivity while maintaining low power consumption and compact dimensions, making them attractive for industrial automation, biomedical detection, intelligent transportation, geomagnetic navigation, and Internet of Things (IoT) applications [20,21,22]. As sensing technologies continue to evolve toward high precision, miniaturization, and intelligent integration [23,24,25,26,27,28,29], the performance of the readout interface has become a key factor limiting the overall sensing accuracy and system reliability. Magnetic sensors constitute an important class of sensing devices that are capable of detecting the magnitude, direction, and temporal variations in magnetic fields. Benefiting from their high sensitivity, non-contact measurement capability, and robust environmental adaptability, magnetic sensors have been extensively deployed in a wide range of applications, including healthcare and biomedical monitoring, precision agriculture and environmental sensing, smart homes, intelligent transportation systems, and industrial automation, as illustrated in Figure 1.

Recent advances in integrated sensing systems have further accelerated the development of high-performance interface circuits for various sensor applications. Precision analog front-ends and dedicated interface ASICs have been extensively investigated for MEMS accelerometers, thermal gas flowmeters, and quartz gyroscopes, where low-noise amplification, high-resolution signal acquisition, and stable reference generation are critical to achieving superior sensing performance [30,31,32,33]. Meanwhile, emerging intelligent sensing platforms increasingly integrate advanced electronic devices, optimization algorithms, and intelligent control strategies to enhance system functionality, reliability, and computational efficiency [34,35,36,37,38]. These developments indicate that modern sensor interface circuits are evolving from simple signal conditioning modules toward highly integrated intelligent sensing systems. Nevertheless, compared with MEMS inertial and flow sensors, the interface circuits for TMR sensors still face greater challenges in simultaneously achieving ultra-low noise, high dynamic range, low offset, rail-to-rail operation, and programmable gain, particularly for weak magnetic signal acquisition. A typical TMR sensor consists of a Wheatstone bridge that generates weak differential output voltages in response to external magnetic fields. However, the bridge output is inevitably affected by process mismatch, large DC offset, thermal noise, and low-frequency flicker noise. These non-idealities substantially degrade the signal-to-noise ratio (SNR), dynamic range, and long-term stability of the sensing system if not properly suppressed. Considerable efforts have been devoted to improving TMR readout circuits in recent years [39,40,41,42,43]. Chopper-stabilized instrumentation amplifiers have been widely adopted to suppress flicker noise and offset voltage, while auto-zeroing and digital calibration techniques have further enhanced offset accuracy. Fully integrated CMOS analog front-ends employing capacitive-feedback amplifiers and low-noise current-mode sensing architectures have demonstrated excellent sensitivity for biomagnetic detection. Moreover, SAR-assisted calibration, ping-pong auto-zeroing techniques, and system-level compensation strategies have significantly improved bandwidth, linearity, and temperature stability for industrial and biomedical applications. Nevertheless, most existing readout circuits are primarily optimized for magnetic field measurement, whereas emerging hardware security applications impose substantially different design requirements.

In practical applications, TMR sensors face significant challenges in signal readout. The differential output of the Wheatstone bridge is typically very weak and is accompanied by substantial DC offset and low-frequency flicker (1/*f*) noise. Direct processing of such signals can severely degrade the dynamic range and consequently deteriorate the overall system performance. Therefore, the design of a high-performance readout circuit capable of accurately converting weak analog magnetic signals into digital information has become a critical challenge in TMR sensor systems.

To address these issues, a dual-operational-amplifier architecture combined with chopper stabilization is adopted to achieve a programmable gain ranging from 1 to 128. To mitigate the large transconductance variation in the input stage over a wide common-mode input range, a transconductance equalization technique is proposed, in which the tail current of the differential input pair is dynamically regulated to maintain an approximately constant overall transconductance across the entire common-mode range. Furthermore, a dynamic slew-rate enhancement technique is introduced by employing a current comparator to detect transient input variations and dynamically inject additional current during large-signal transitions, thereby significantly improving the slew rate with negligible increase in static power consumption. In addition, a digitally assisted offset calibration circuit is designed using a programmable current-source array to finely compensate the input offset, resulting in a substantial reduction in the equivalent input-referred offset voltage.

## 2. Proposed Programmable Gain Instrumentation Amplifier

**Figure 2:**
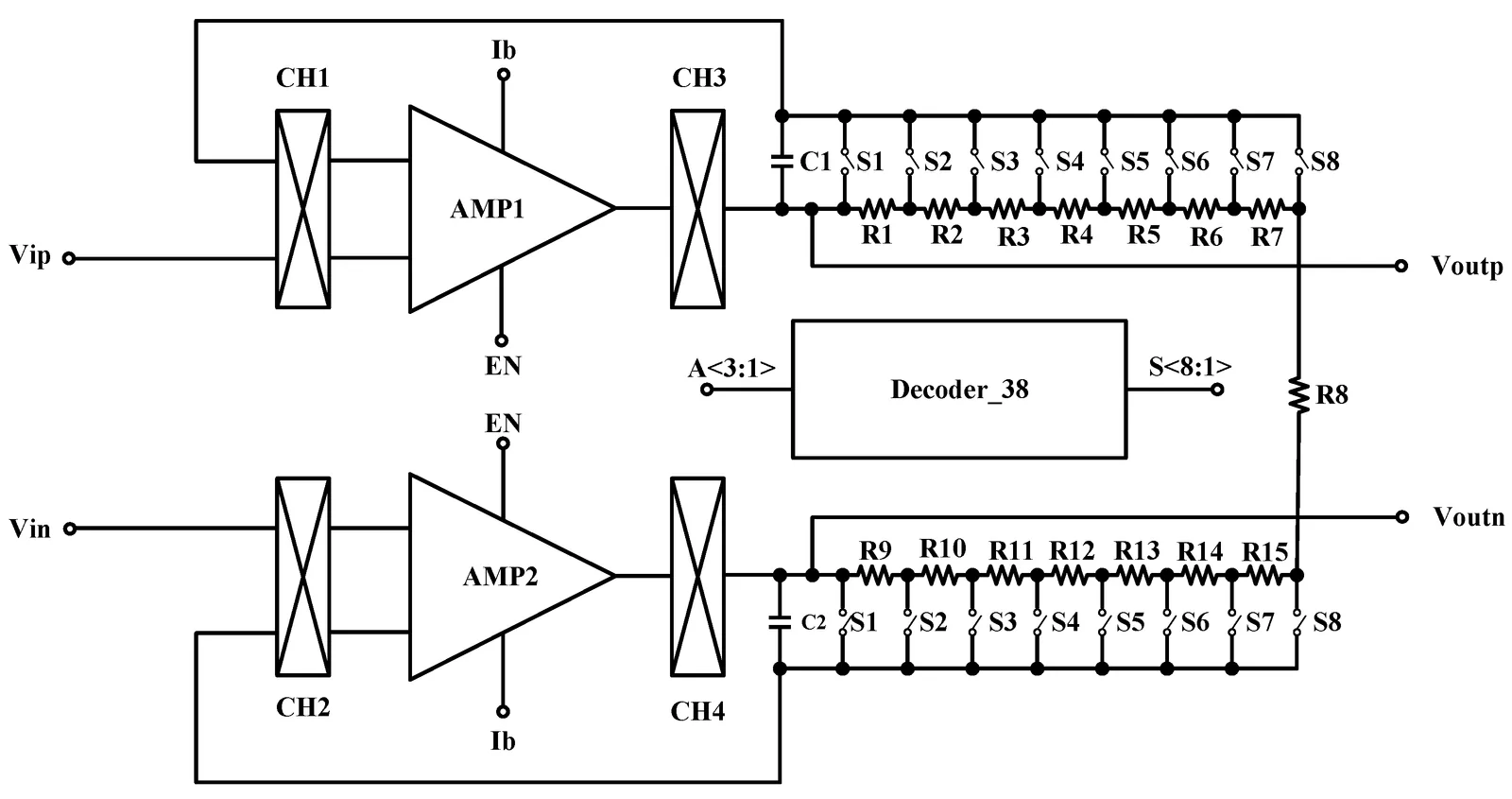
Architecture of the proposed chopper-stabilized programmable gain instrumentation amplifier (PGIA).

Figure 2 illustrates the architecture of the proposed chopper-stabilized programmable gain instrumentation amplifier (PGIA). The circuit adopts a dual-operational-amplifier topology consisting of a 3-to-8 decoder, chopper switches, and a programmable instrumentation amplifier. The decoder converts a 3-bit digital control word into eight mutually exclusive control signals, which are used to configure the gain of the programmable amplifier. The differential input signals, *V_IP_* and *V_IN_*, are first modulated by the input chopper and then amplified by the instrumentation amplifier. Through the chopper modulation process, the input-referred offset voltage and low-frequency flicker noise are translated to the chopping frequency, whereas the desired signal is subsequently demodulated back to baseband at the amplifier output.

The signal spectrum is separated from the offset and low-frequency noise components, effectively suppressing the input offset voltage and 1/*f* noise. The amplified differential signal is then processed by the programmable gain stage. Eight digital control signals generated by the decoder configure the resistor network to provide programmable gains ranging from 1 to 128. As a result, the proposed PGIA simultaneously achieves wide gain programmability together with low offset and low input-referred noise. The programmable gain stage employs a dual-amplifier topology with tapped resistor arrays. Since the closed-loop gain is directly determined by the feedback resistance, resistor matching is critical for achieving high gain accuracy. Therefore, all resistors are implemented using identical unit resistor cells, while different resistance values are realized by adjusting the number of series-connected unit elements rather than changing their physical dimensions. This layout strategy significantly improves matching accuracy and minimizes process-induced gain variation.

A tapped resistor array is adopted instead of placing switches directly in the signal path. If the switches were connected in series with the feedback resistors, their on-resistance would introduce additional gain errors. In the proposed architecture, the switches are connected to resistor taps, allowing the feedback resistance to remain unaffected by the switch resistance and thereby improving gain accuracy. Furthermore, compensation capacitors *C*_1_ and *C*_2_ are connected across the resistor arrays to introduce a feedforward path, which improves loop stability and extends the high-frequency response.

**A.**  **Chopper-Stabilized Operational Amplifier**

**Figure 3:**
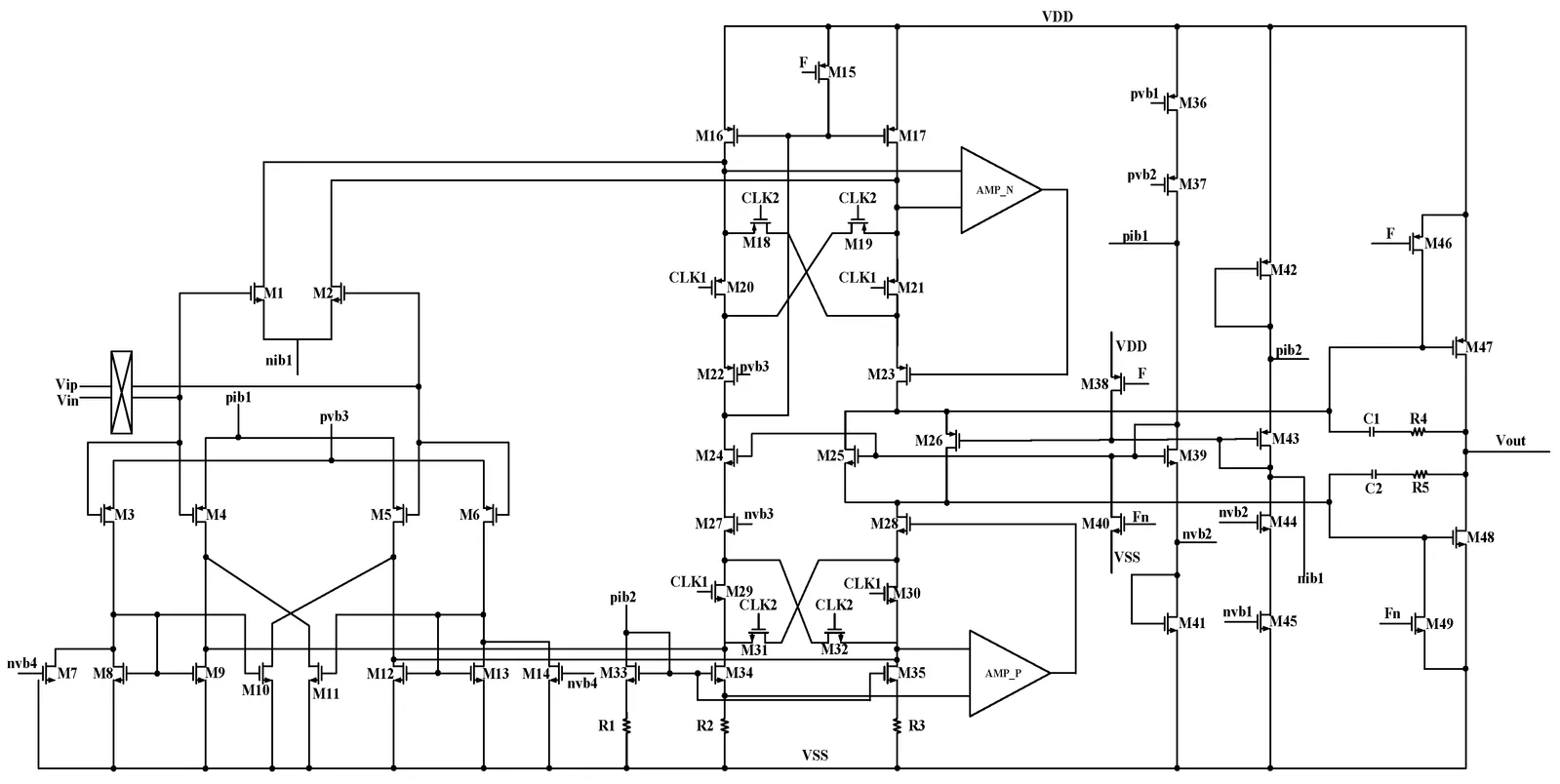
Architecture of the proposed chopper operational amplifier.

The proposed chopper-stabilized operational amplifier consists of a bias circuit, input and output chopper switches, a rail-to-rail complementary input stage, a folded-cascode first stage, and a Class-AB output stage, as illustrated in Figure 3. The amplifier adopts a two-stage architecture, where the folded-cascode first stage provides a high output impedance to achieve a high open-loop gain, while the second-stage Class-AB output stage offers additional voltage gain together with a large output swing, thereby enabling rail-to-rail output operation.

To suppress the input-referred offset voltage and low-frequency flicker noise, a pair of input chopper switches is placed ahead of the differential input stage to modulate the input signal, offset, and 1/*f* noise from the baseband to the chopping frequency. The output chopper switches are inserted at the folded current-mirror nodes of the first-stage folded-cascode amplifier, where the amplified high-frequency signal is synchronously demodulated back to the baseband. Although chopper modulation inevitably introduces a certain reduction in the open-loop gain, locating the output chopper switches at the folded nodes confines the gain degradation primarily to the input differential pair while leaving the gain contribution of the folded-cascode stage essentially unaffected. Consequently, the overall open-loop gain is only slightly reduced, leading to a smaller closed-loop gain error and improved gain accuracy. The chopping frequency was selected as 20 kHz, which is considerably higher than the useful low-frequency magnetic-signal band and the dominant flicker-noise corner. This selection provides a suitable compromise among low-frequency noise suppression, switching ripple, amplifier bandwidth, and power consumption. After demodulation, a second-order Butterworth active low-pass filter is employed to suppress the up-modulated offset, flicker noise, and switching components around the chopping frequency. The filter has a cutoff frequency and −3 dB bandwidth of 500 Hz, with a quality factor of 0.707 and a roll-off rate of −40 dB/dec. Its transfer function is expressed as

(1) $$H\left(s\right)=\frac{\omega_{c}^{2}}{s^{2}+\sqrt{2}\omega_{c}s+\omega_{c}^{2}},\omega_{c}=2\pi \times \frac{500\;rad}{s}\;\;\;$$

Since the ratio between the chopping frequency and the filter cutoff frequency is 40, the calculated attenuation at the chopping frequency is approximately 64.1 dB. Thus, the increased noise and switching ripple around 20 kHz are effectively removed without attenuating the useful low-frequency magnetic-field signals. The measured residual magnetic-noise density after the complete signal-conditioning chain is 0.13 nT/$\sqrt{Hz}$ at 1 Hz.

The input stage employs complementary NMOS and PMOS differential pairs connected in parallel, allowing the common-mode input voltage range to extend from ground to *V*_DD_, thereby realizing true rail-to-rail input operation. The first amplification stage adopts a folded-cascode topology to simultaneously achieve high DC gain and wide input common-mode range. The second stage is implemented using a floating-current-source-biased Class-AB output stage, which accurately establishes the quiescent bias current while providing a large output driving capability and a rail-to-rail output voltage swing. By combining chopper stabilization with the proposed rail-to-rail folded-cascode architecture and the Class-AB output stage, the operational amplifier simultaneously achieves low offset, low flicker noise, high gain, and wide input/output dynamic range, making it well suited for high-resolution sensor interface applications.

**B.**  **Bias Circuit**

**Figure 4:**
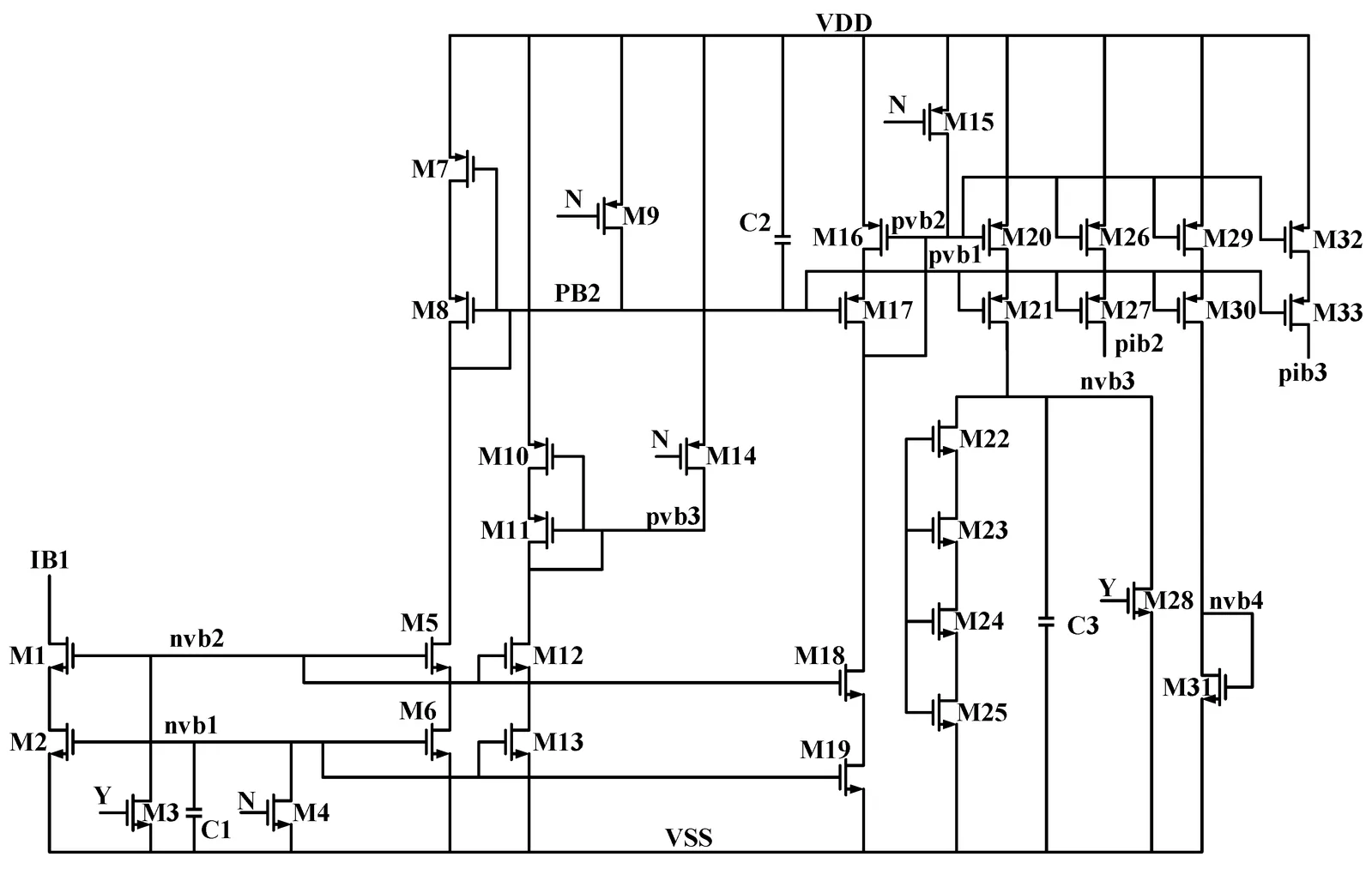
Schematic of the proposed bias circuit.

A stable and low-noise bias network is essential for guaranteeing the performance consistency of the proposed chopper-stabilized PGIA. The schematic of the bias circuit is shown in Figure 4. The proposed bias generator is implemented using cascoded current mirrors to generate multiple bias voltages and bias currents required by the rail-to-rail input stage, folded-cascode amplifier, and Class-AB output stage.

The bias circuit starts from a reference current *I*_B1_, which is replicated through a hierarchy of NMOS and PMOS current mirrors. The lower NMOS mirror branches generate four bias voltages, denoted as nvb_1_, nvb_2_, nvb_3_, and nvb_4_, while the upper PMOS mirror branches produce the corresponding PMOS bias voltages pvb_1_, pvb_2_, and pvb_3_. Additional cascoded mirror branches further generate the bias currents pib_2_ and pib_3_, which are distributed to different amplifier stages. Each bias node serves a specific function within the amplifier. The bias voltage pvb_1_ establishes the gate bias of the PMOS folded-cascode devices, ensuring that they remain in saturation under all operating conditions. The voltage pvb_2_ determines the quiescent current of the Class-AB output stage, thereby balancing power consumption and output driving capability. The current pib_2_ biases the intermediate current mirrors, whereas nvb_1_ provides the reference current for the lower output branch. The voltages nvb_2_ and nvb_3_ determine the operating points of the intermediate gain stage and the folded differential pair, respectively, while nvb_4_ supplies the tail current of the complementary input differential pairs to ensure stable transconductance over the entire common-mode input range.

Since the proposed amplifier employs chopper modulation, periodic switching activity may introduce ripple into the bias nodes through parasitic coupling. Such ripple directly modulates the bias currents and deteriorates the amplifier noise performance. To suppress this effect, three large decoupling capacitors are connected to the critical bias nodes. These capacitors act as low-pass filters that significantly attenuate the ripple generated by the chopping clocks, thereby stabilizing the mirror currents and improving both offset and noise performance.

Compared with a conventional bias generator, the proposed cascoded architecture provides higher output impedance and superior current-copy accuracy while maintaining excellent immunity to supply-voltage fluctuations. Consequently, all amplifier stages operate under well-controlled bias conditions, which contributes to the stability and robustness of the entire PGIA.

**C.**  **Digital Gain Control Circuit**

To realize programmable gain operation, a digital gain control circuit based on a 3-to-8 decoder is incorporated into the PGIA. The decoder converts a 3-bit digital control word into eight mutually exclusive output signals, each of which controls one gain-selection switch in the feedback resistor network. The decoder is implemented entirely using static CMOS logic gates to minimize static power consumption while ensuring reliable digital operation. NAND gates are adopted as the fundamental building blocks because of their compact implementation and excellent driving capability. According to the truth table shown in Table 1, only one decoder output is asserted for each digital input code, thereby enabling a single gain path at any given time.

The three-bit control word *A*<2:0> corresponds to eight programmable gain settings:

(2) G={1, 2, 4, 8, 16, 32, 64, 128} V/V

When the input code changes, the corresponding decoder output activates one switch within the tapped resistor array while all remaining switches remain disabled. Consequently, the effective feedback resistance is reconfigured, leading to the desired closed-loop gain. Unlike resistor-string architectures employing switches directly in the signal path, the proposed decoder only controls the resistor taps, thereby preventing the switch on-resistance from affecting the effective feedback resistance. This architecture significantly improves gain accuracy while simplifying digital control logic. Because only one output is active at any time, the decoder exhibits negligible dynamic power consumption under static operating conditions. Moreover, the digital control interface is fully compatible with standard CMOS logic, allowing straightforward integration into mixed-signal systems.

## 3. Circuit Optimization Techniques

Rail-to-rail input operation using complementary NMOS and PMOS differential pairs is a well-established technique. In a conventional complementary input stage, only the PMOS pair operates near the negative supply rail, only the NMOS pair operates near the positive supply rail, and both pairs conduct in the intermediate common-mode region. Consequently, the total input transconductance varies considerably with the input common-mode voltage. Assuming strong-inversion operation, the total transconductance can be expressed approximately as

(3) $$g_{m,sum}=\sqrt{2\mu_{n}C_{ox}{\left(\frac{W}{L}\right)}_{n}I_{n}}+\sqrt{2\mu_{p}C_{ox}{\left(\frac{W}{L}\right)}_{p}I_{p}}$$

Therefore, maintaining a constant $g_{m,sum}$ requires coordinated control of the two tail currents $I_{n}$ and $I_{p}$ during the transition between the NMOS- and PMOS- dominated operating regions.

Several bias-current control techniques have been reported. A representative approach uses current switches and three-times current mirrors to increase the tail current of the remaining active differential pair from I to 4I when the complementary pair is turned off [34]. Although this method compensates for the transconductance reduction in the inactive pair, it requires high-ratio current mirrors and can produce residual $g_{m}$ variation during current handover. Another approach employs DC level shifters to overlap the transition regions of the NMOS and PMOS pairs [35]. This technique achieves a smoother transconductance characteristic but introduces additional level-shifting and bias circuits and depends on accurate alignment of the two transition regions.

**Figure 5:**
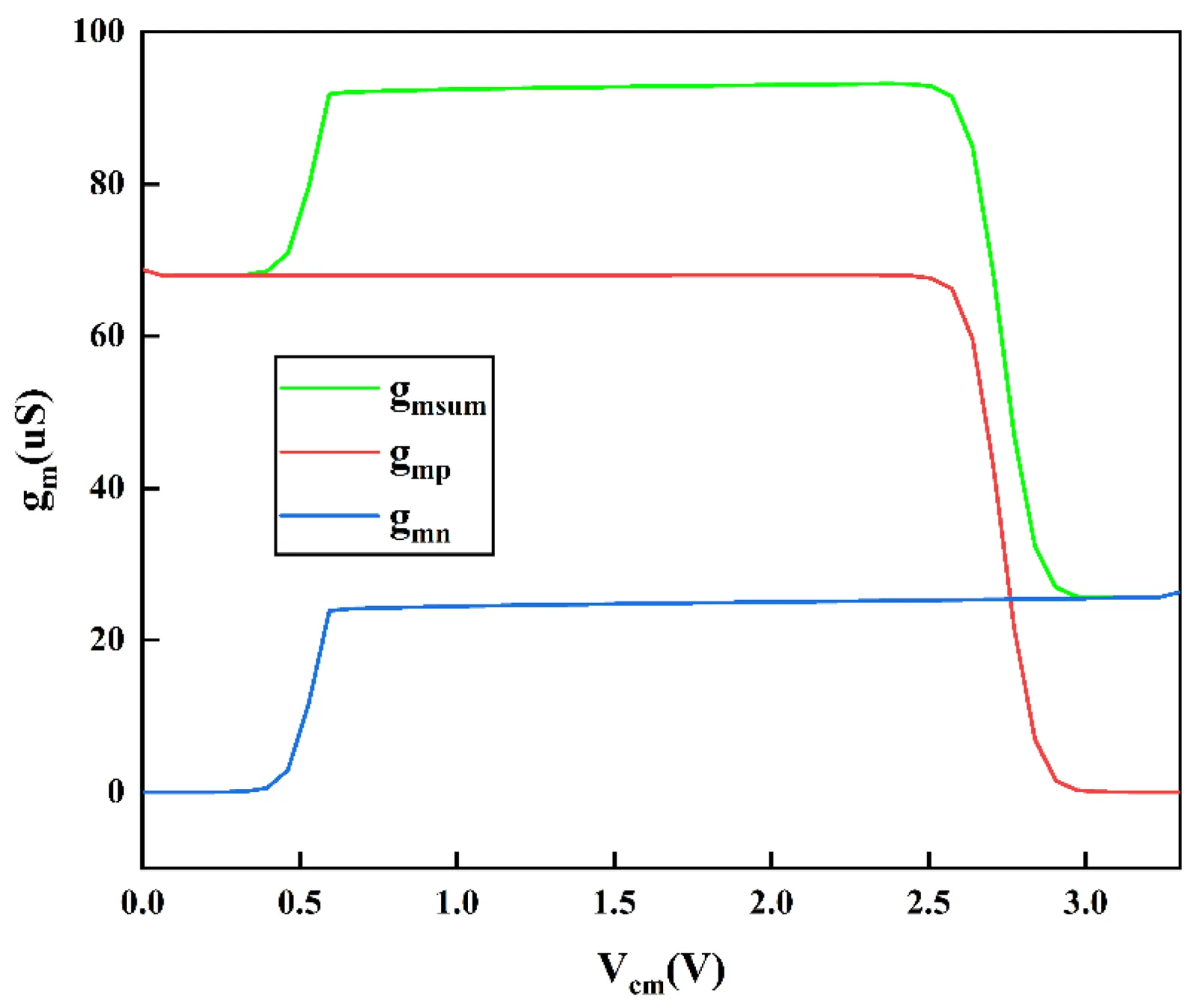
Transconductance characteristics of the conventional complementary input stage.

Although complementary NMOS and PMOS differential input pairs provide rail-to-rail common-mode operation, their transconductance characteristics vary significantly with the input common-mode voltage. As illustrated in Figure 5, a conventional complementary input stage exhibits a pronounced hump-shaped transconductance profile because the NMOS and PMOS differential pairs dominate in different common-mode regions. Consequently, the total input transconductance may vary by more than a factor of two across the input range.

**Figure 6:**
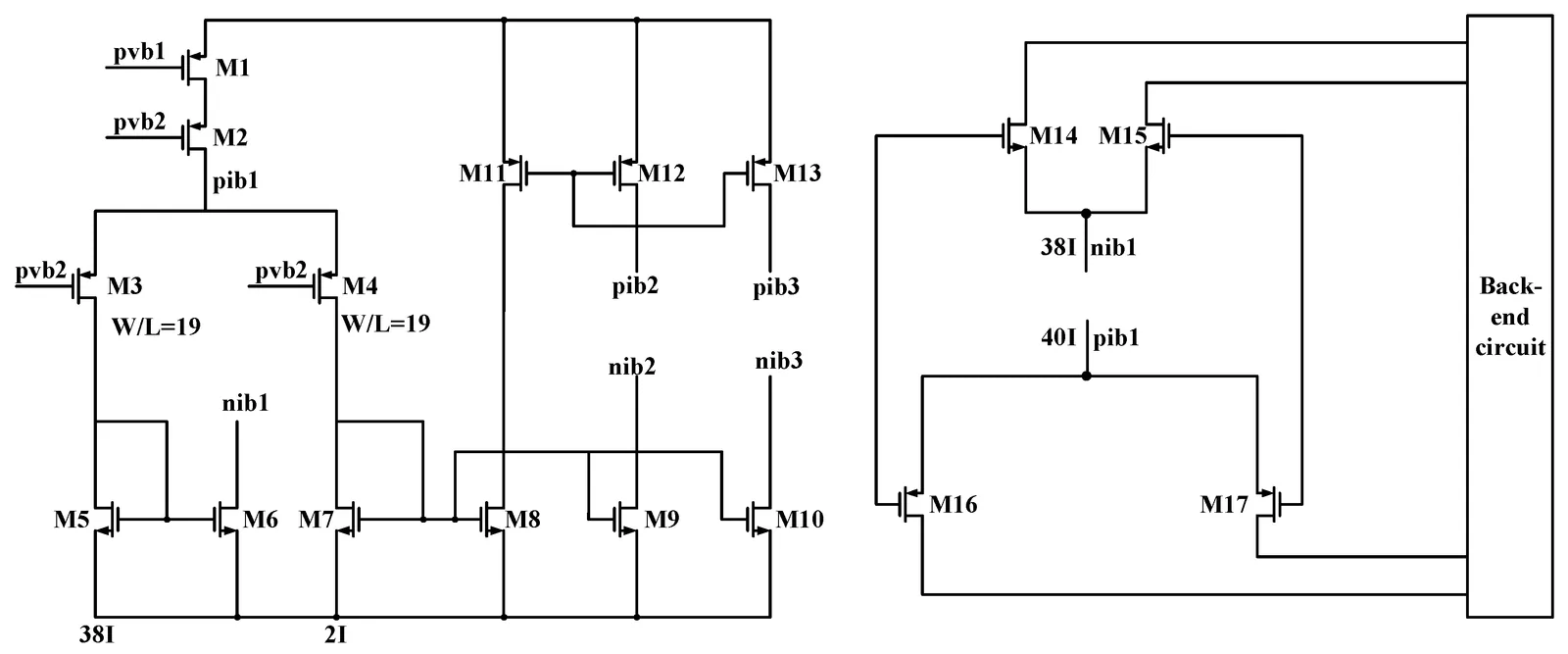
Schematic of the proposed transconductance equalization circuit.

Such transconductance variation directly affects several important amplifier characteristics, including the gain-bandwidth product (GBW), input-referred noise, offset voltage, and closed-loop gain accuracy. Therefore, maintaining a nearly constant total transconductance throughout the entire common-mode range is essential for achieving stable amplifier performance. To address this issue, a dynamic transconductance equalization circuit is proposed, as shown in Figure 6. The circuit consists of two switching transistors and several current mirrors that automatically redistribute the bias current between the NMOS and PMOS differential pairs according to the input common-mode voltage.

Unlike conventional schemes employing a dedicated common-mode detector or DC level-shifting circuits, the proposed transconductance-equalization circuit directly utilizes the conduction states of the complementary input pairs to control the bias-current redistribution. As shown in Figure 6, two switching transistors and the associated current mirrors redistribute the available bias current between the NMOS and PMOS input pairs. When only one input pair remains active, approximately 95% of the available current is directed to that pair, whereas the remaining current is supplied to the auxiliary linearization branch. The current-mirror ratios are determined by considering the mobility difference and device aspect ratios of the NMOS and PMOS input transistors.

This implementation avoids a separate common-mode sensing amplifier and additional DC level shifters. Moreover, no additional devices are inserted in series with the signal path of the input differential pairs. The proposed circuit is therefore suitable for integration with the chopper-stabilized folded-cascode input stage, where preserving input headroom and limiting additional input-referred noise are important design considerations.

When the common-mode voltage approaches ground, only the PMOS differential pair remains active, whereas the NMOS pair is completely turned off. Under this condition, the total transconductance is solely determined by the PMOS input devices,

(4) $$g_{m,sum}=g_{mp}$$

As the common-mode voltage increases into the middle operating region, both differential pairs conduct simultaneously. The proposed current mirrors dynamically adjust the bias currents of the two input stages, resulting in

(5) $$\;\;\;\;g_{m,sum}=g_{mn}+g_{mp}=\sqrt{2\mu_{n}C_{ox}{\left(\frac{W}{L}\right)}_{n}\cdot 8I_{1}}+\sqrt{2\mu_{P}C_{ox}{\left(\frac{W}{L}\right)}_{P}\cdot 8I_{2}}$$

Instead of allowing both transconductances to increase independently, the current redistribution network gradually decreases the PMOS bias current while proportionally increasing the NMOS bias current. Consequently, the increase in one differential pair compensates for the decrease in the other, producing a nearly constant total transconductance.

When the common-mode voltage approaches the positive supply, the PMOS differential pair gradually turns off, whereas the NMOS differential pair becomes dominant. At this stage, the switching transistors activate an auxiliary current mirror that redistributes the tail current. By properly selecting the transistor aspect ratios, approximately 95% of the bias current is injected into the NMOS input stage, while only a small fraction is supplied to the auxiliary transconductance linearization loop. As a result,

(6) $$g_{m,sum}=g_{mn}$$

Assuming that

(7) $$\mu_{n}C_{ox}{\left(\frac{W}{L}\right)}_{n}=\mu_{p}C_{ox}{\left(\frac{W}{L}\right)}_{p}$$

the proposed current allocation strategy maintains an approximately constant total transconductance over the entire common-mode input range.

**Figure 7:**
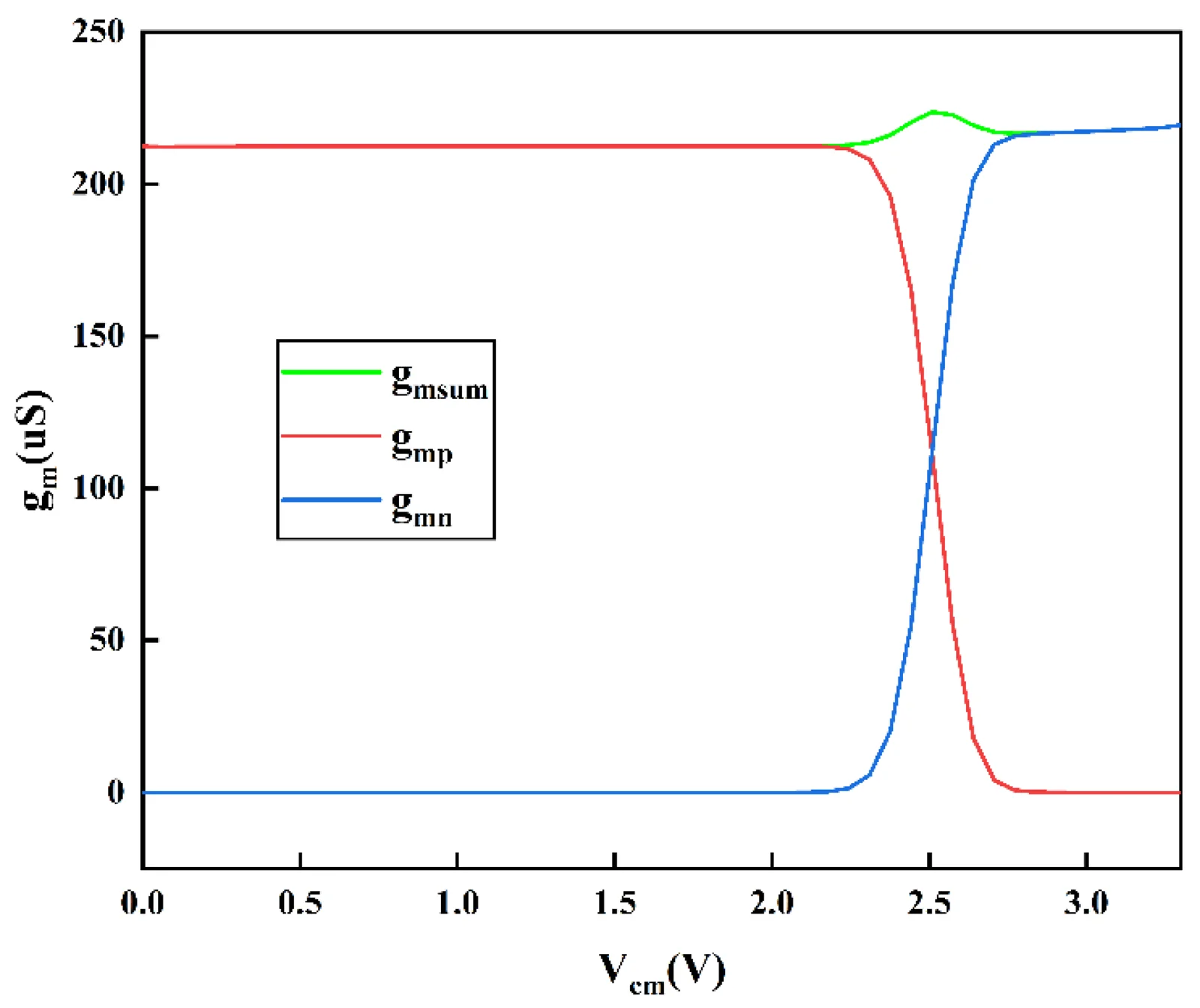
Overall input transconductance characteristics with the proposed transconductance equalization technique.

Simulation results shown in Figure 7 confirm the effectiveness of the proposed technique. Compared with the conventional complementary input stage, the variation in the overall transconductance is dramatically reduced, while the gain-bandwidth product remains nearly constant throughout the full rail-to-rail input range. Furthermore, the stabilized transconductance improves input-referred noise, enhances gain consistency among different common-mode voltages, and reduces closed-loop gain variation at all programmable gain settings.

To quantitatively evaluate the proposed technique, the total input transconductance was simulated over the complete 0–3.3 V common-mode input range. As shown in Figure 7, the total transconductance of the uncompensated complementary input stage varies by more than a factor of two. After applying the proposed current-redistribution circuit, the total transconductance is maintained within approximately 211–224 μS, corresponding to a peak-to-peak variation of approximately 6%, as shown in Figure 7. The proposed technique therefore substantially reduces the common-mode dependence of the input transconductance. This stabilization also improves the consistency of the gain-bandwidth product, input-referred noise, and closed-loop gain over the rail-to-rail input range. The comparison of bias-current control techniques for rail-to-rail input stages is shown in Table 2.

## 4. Simulation Results

To verify the effectiveness of the proposed programmable gain instrumentation amplifier (PGIA), extensive post-layout simulations were carried out using the SMIC 0.18 μm CMOS process under a 3.3 V power supply. The proposed design was evaluated in terms of frequency response, transient performance, noise characteristics, and offset calibration capability. The simulation results demonstrate that the proposed optimization techniques significantly improve the overall amplifier performance while maintaining low power consumption.

**A.**  **Frequency Response**

**Figure 8:**
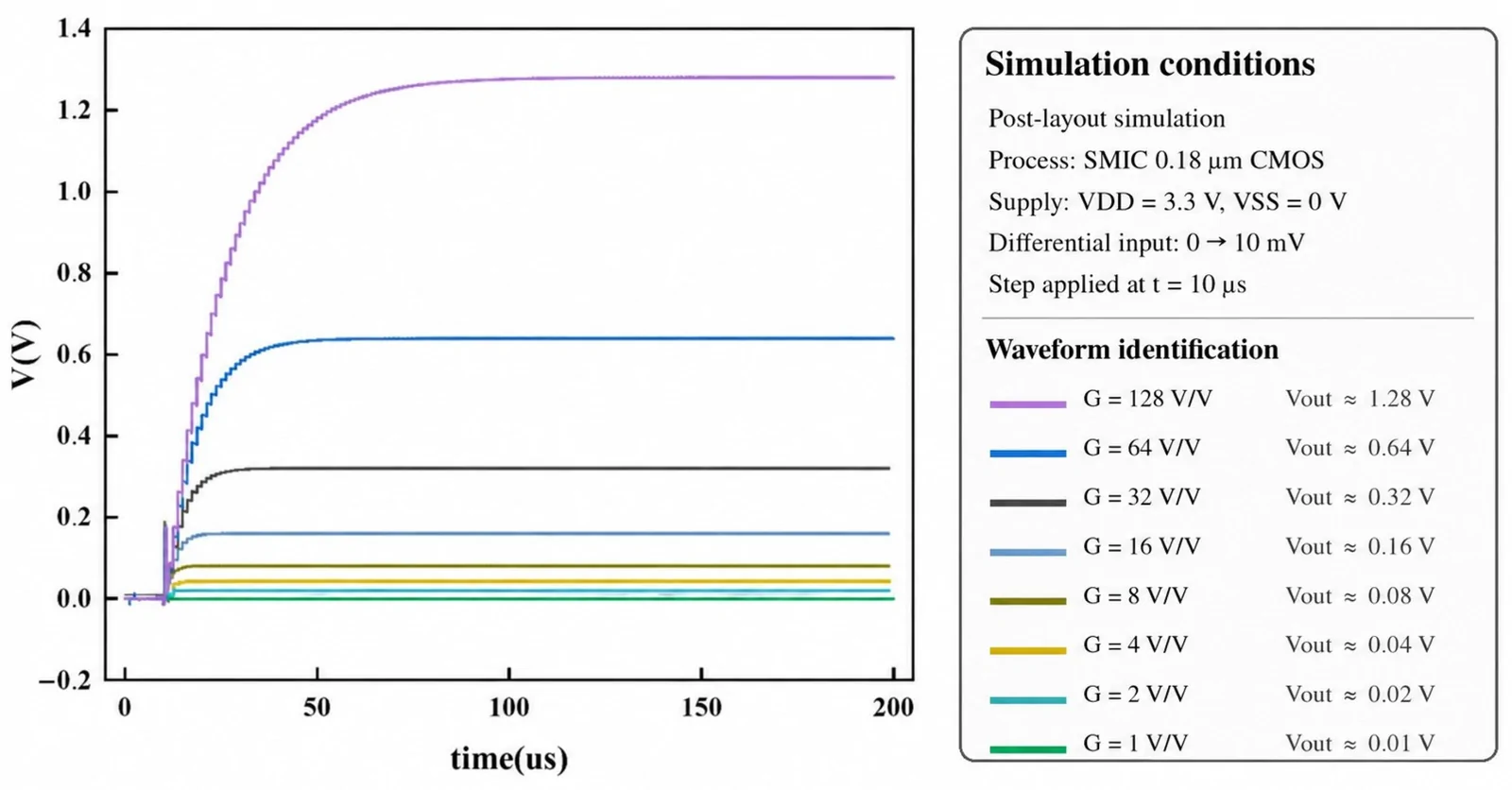
Post-layout transient output responses of the proposed PGIA under different programmable gain settings. Simulation conditions: SMIC 0.18 μm CMOS process, $V_{DD}=3.3\;V$, $V_{SS}=0\;V$, and a differential input step from 0 to 10 mV applied at t=10 μs. The eight curves correspond to gains of 1, 2, 4, 8, 16, 32, 64, and 128 V/V.

Figure 8 presents the post-layout transient output responses of the proposed PGIA under different programmable gain settings. The simulations were performed using the SMIC 0.18 μm CMOS process with $V_{DD}=3.3\;V$ and $V_{SS}=0\;V$. A differential input step from 0 to 10 mV was applied at t=10μs. The closed-loop gain was successively configured as 1, 2, 4, 8, 16, 32, 64, and 128 V/V, resulting in steady-state output voltages of approximately 0.01, 0.02, 0.04, 0.08, 0.16, 0.32, 0.64, and 1.28 V, respectively. The results confirm that the proposed programmable resistor network provides the intended gain settings and stable transient output responses over the entire programmable-gain range.

Since the amplifier employs a digitally programmable resistor network, the closed-loop gain can be configured from 1 V/V to 128 V/V through the 3-bit digital control word. As expected, the closed-loop bandwidth decreases as the programmed gain increases, following the classical gain-bandwidth trade-off of feedback amplifiers. At low gain settings, the amplifier provides a relatively wide signal bandwidth, making it suitable for high-speed signal acquisition. As the gain increases, the feedback factor decreases, resulting in a reduced closed-loop bandwidth while maintaining sufficient gain accuracy for precision sensor applications. More importantly, the gain-bandwidth product (GBW) remains nearly constant over the entire programmable gain range, indicating that the proposed transconductance equalization technique effectively stabilizes the input-stage transconductance. Consequently, the amplifier exhibits highly consistent dynamic characteristics regardless of the selected gain. In addition, all gain configurations exhibit adequate phase margins without noticeable gain peaking, demonstrating that the proposed Miller compensation together with feedforward compensation successfully guarantees loop stability throughout the entire operating range. These results verify that the proposed programmable resistor network and frequency-compensation strategy simultaneously achieve wide gain programmability and stable closed-loop operation.

**B.**  **Transient Response**

**Figure 9:**
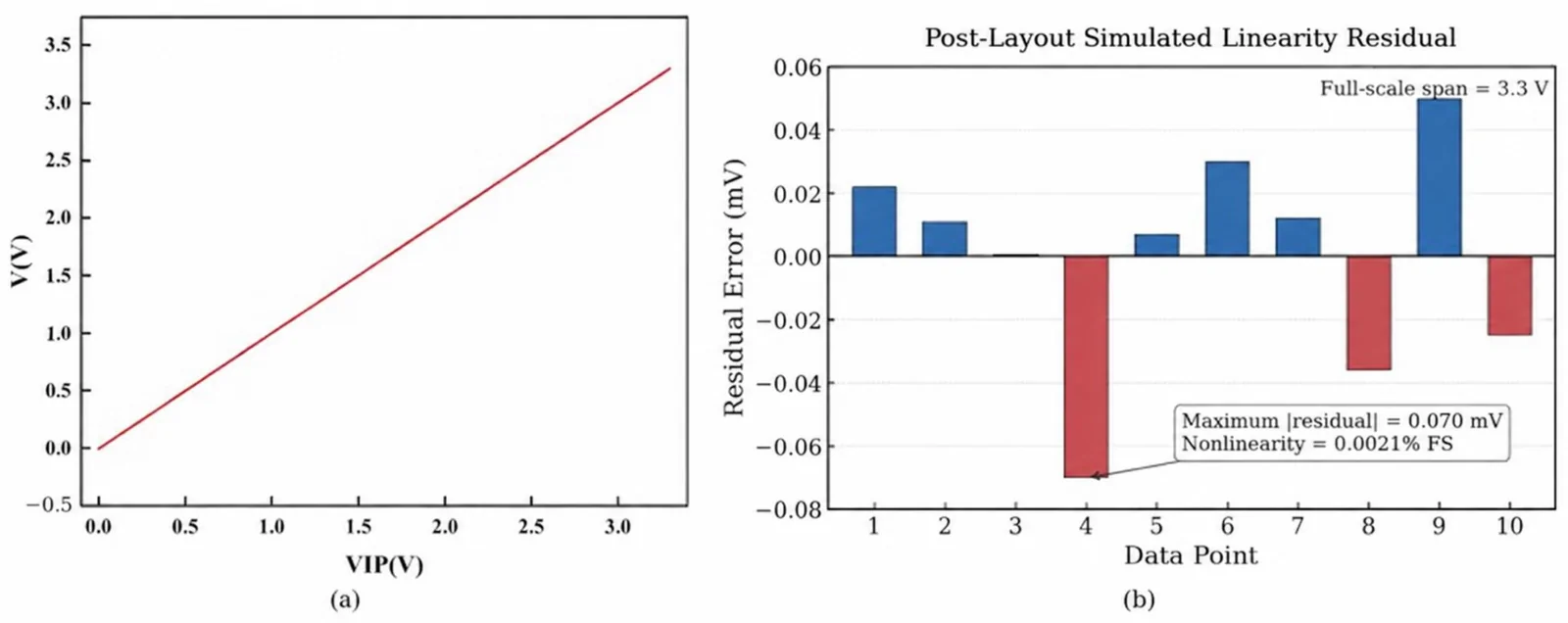
(**a**) Post-layout simulated large-signal step responses of the PGIA with the dynamic slew-rate enhancement circuit enabled and disabled at a closed-loop gain of 1 V/V, a load capacitance of 20 pF, and a 3.3 V supply. (**b**) The error in the measured linearity.

The transient simulation results are presented in Figure 9, where the proposed amplifier is excited by a large differential input step to evaluate its large-signal behavior. Without the dynamic slew-rate enhancement circuit, the charging and discharging currents of the Miller compensation capacitor are limited by the quiescent bias current, resulting in relatively slow output transitions and prolonged settling time. Consequently, both the rising and falling edges become significantly steeper, indicating a substantial improvement in the slew rate. Because the auxiliary current is generated only during transient operation, the quiescent current remains unchanged once the amplifier returns to steady-state conditions. Therefore, the proposed architecture successfully improves transient response without increasing static power consumption. Another important observation is that the output waveform exhibits negligible overshoot and ringing after the large-signal transition. This behavior confirms that the additional transient current does not degrade the loop stability established by the compensation network. Instead, the proposed circuit accelerates the charging and discharging processes of the compensation capacitor while preserving the original small-signal characteristics of the amplifier. These results demonstrate that the proposed slew-rate enhancement technique effectively resolves the traditional trade-off between transient speed and power efficiency, making the amplifier particularly suitable for applications requiring both high precision and rapid signal acquisition.

**C.**  **Noise Performance**

**Figure 10:**
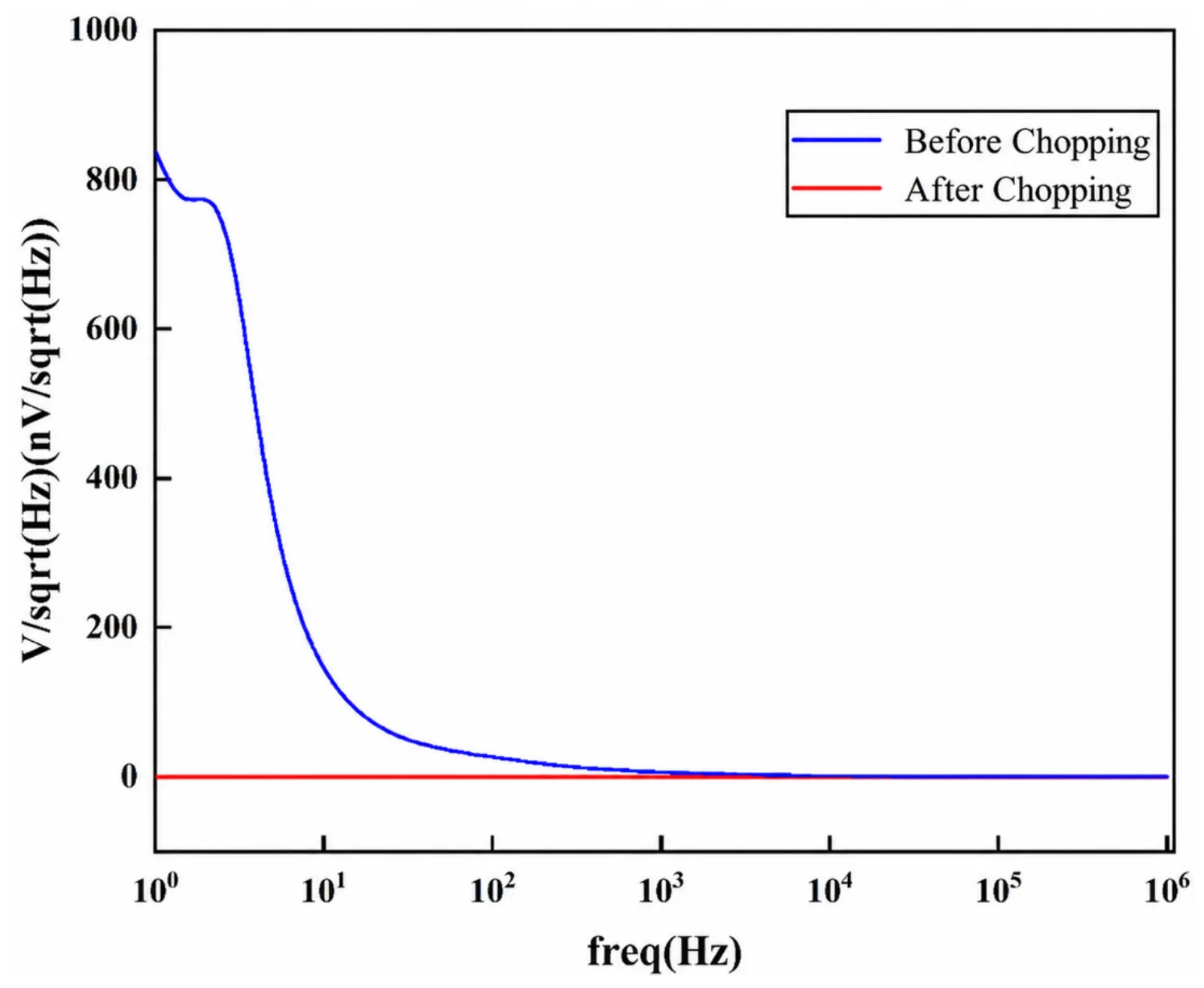
Simulated input-referred noise power spectral density (PSD) of the proposed PGIA.

The simulated input-referred noise spectrum of the proposed amplifier is shown in Figure 10. Owing to the combined effects of chopper stabilization and the low-noise folded-cascode architecture, the amplifier exhibits excellent low-frequency noise performance. At frequencies below the chopping frequency, the conventional 1/*f* noise is effectively suppressed because the chopper modulation translates the flicker noise to higher frequencies. After demodulation, only the desired signal is restored to baseband, whereas the low-frequency noise components remain outside the signal bandwidth and can be removed by the following low-pass filtering process. Consequently, the input-referred noise spectrum is dominated by thermal noise over most of the operating frequency range, resulting in a significantly flatter noise profile than that of conventional instrumentation amplifiers. Furthermore, the proposed transconductance equalization technique maintains nearly constant input transconductance across the entire common-mode input range. Since the equivalent thermal noise is inversely proportional to the input transconductance, stabilizing the transconductance also improves the consistency of the input-referred noise under different operating conditions. The simulation results therefore confirm that the proposed amplifier simultaneously achieves low flicker noise, low thermal noise, and stable noise characteristics over a wide common-mode input range, making it highly suitable for high-resolution biomedical and sensor interface applications.

## 5. Results and Discussion

**A.**  **Experimental Setup**

**Figure 11:**
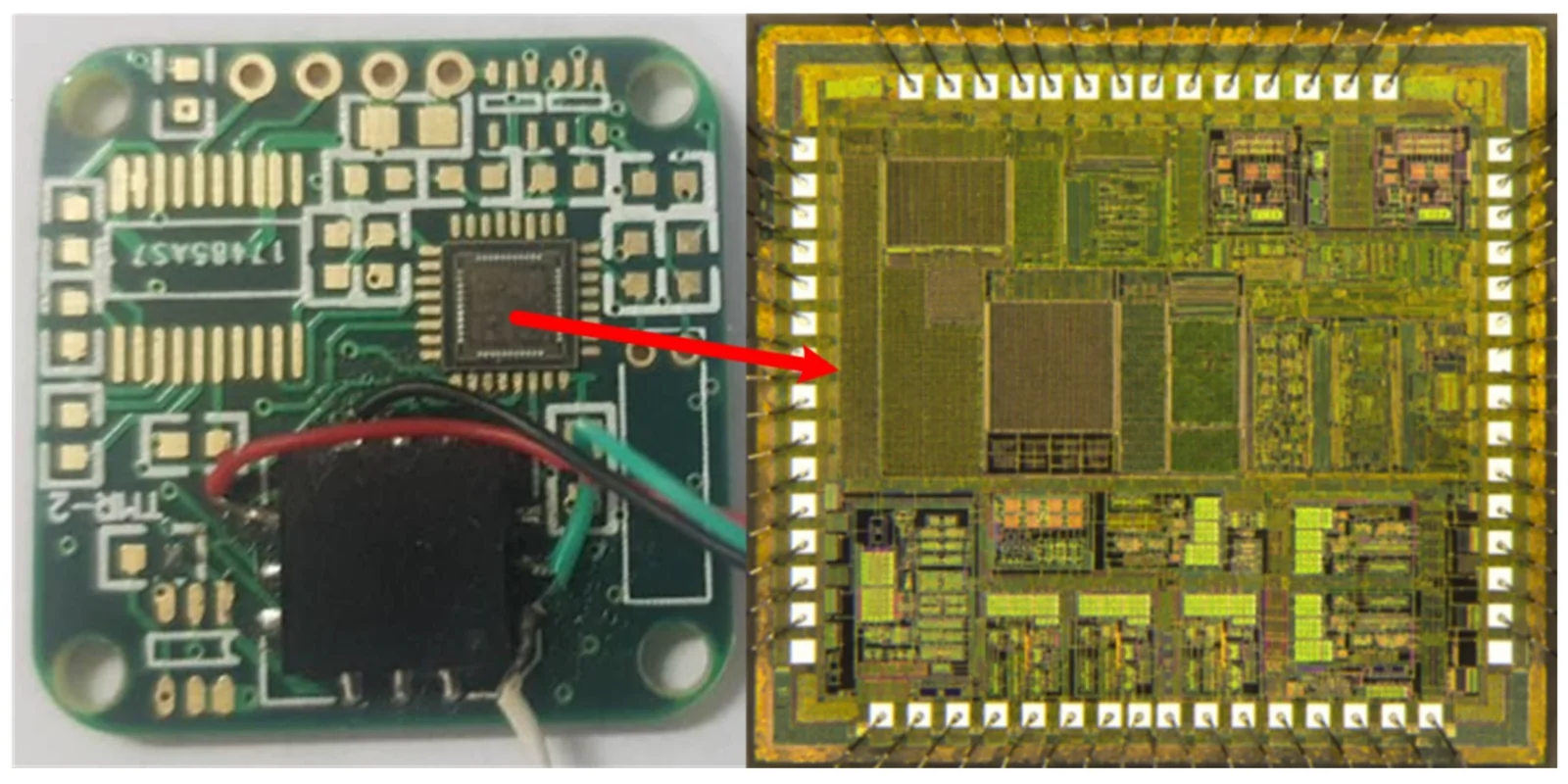
Photograph of the fabricated PCB and ASIC chip.

The proposed TMR sensor (Multidimension Technologies, Suzhou, China) interface ASIC was fabricated using the standard SMIC 0.18 μm CMOS process. The fabricated die was mounted on a four-layer printed circuit board (PCB) and wire-bonded using aluminum bonding wires to establish electrical connections between the chip pads and the PCB. The interface ASIC was then integrated with a TMR magnetic sensor probe to form a compact TMR magnetometer prototype, as shown in Figure 11. The magnetometer prototype was powered from a single external 5 V supply. An on-board low-dropout regulator generated a regulated 3.3 V rail for the readout ASIC. The PGIA, chopper switches, bias generator, gain-control decoder, offset-calibration circuit, and associated digital control logic were all operated from this 3.3 V rail. The TMR Wheatstone bridge was directly biased from the 5 V supply, whereas the ADC, microcontroller, and communication interface operated at 3.3 V. Therefore, the 3.3 V supply adopted in the post-layout simulations was consistent with the actual operating voltage of the ASIC core. Unless otherwise stated, the reported power consumption of 10 mW includes the readout ASIC and the TMR sensing bridge but excludes the external PC, RS-232 converter, Helmholtz-coil driver, and reference fluxgate magnetometer.

**Figure 12:**
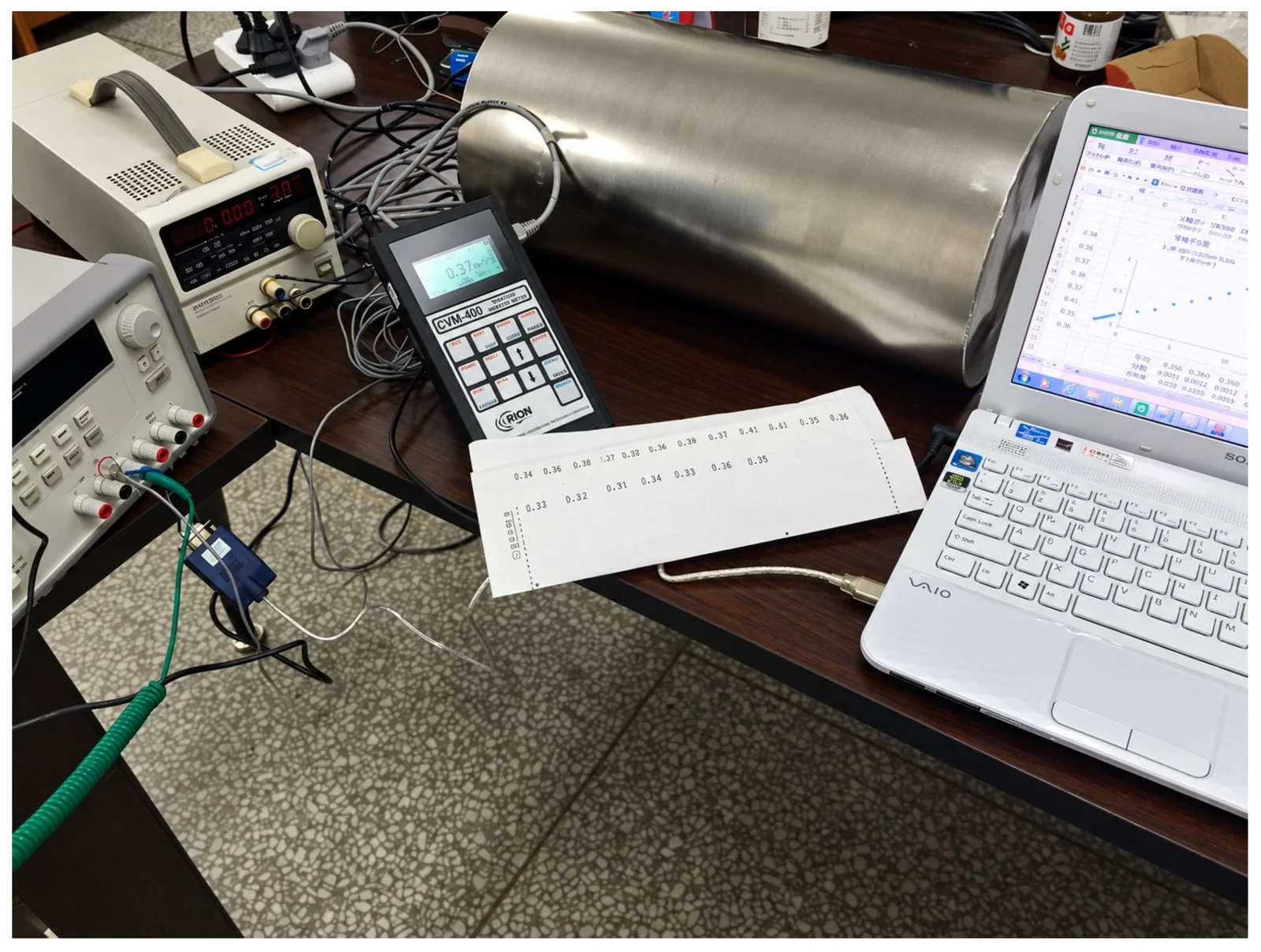
Experimental platform for magnetic-field measurements.

To evaluate the performance of the proposed interface circuit under a well-controlled magnetic environment, a dedicated magnetic measurement platform was established as shown in Figure 12. Since ambient magnetic interference generated by surrounding electrical equipment may reach several tens of micro-T, it is difficult to accurately characterize a high-sensitivity TMR sensor under ordinary laboratory conditions. Therefore, all measurements were carried out inside a three-layer permalloy magnetic shielding chamber (Jiangsu MultiDimension Technology Co., Ltd., Suzhou, China), whose residual magnetic field at the geometric center is approximately 1 nT.

A pair of Helmholtz coils was placed inside the shielding chamber to generate a uniform magnetic field. The excitation current of the Helmholtz coils was supplied by a Kenwood PW36-1.5ADP precision current source, while the generated magnetic field was calibrated using a high-precision FVM-400 fluxgate magnetometer. During measurement, the sensitive axes of both the TMR sensor and the FVM-400 probe were carefully aligned and positioned as closely as possible to ensure that both devices experienced the same magnetic field. The digital output of the proposed interface ASIC was transmitted to a PC through an RS-232 interface operating at 9600 bps for real-time data acquisition and analysis.

**B.**  **Linearity Measurement**

To evaluate the linearity of the proposed TMR magnetometer system, the magnetic field generated by the Helmholtz coils was gradually adjusted by controlling the output current of the Kenwood PW36-1.5ADP precision current source. The corresponding magnetic field intensity was simultaneously calibrated using the FVM-400 fluxgate magnetometer as the reference standard. The applied magnetic field was swept from −100 μT to +100 μT. Eleven equally spaced measurement points were selected with an interval of 20 μT, together with an additional measurement point at 0 μT. At each magnetic field level, the digital output of the proposed TMR interface ASIC was recorded through the RS-232 interface, while the corresponding reference magnetic field measured by the FVM-400 was simultaneously collected.

**Figure 13:**
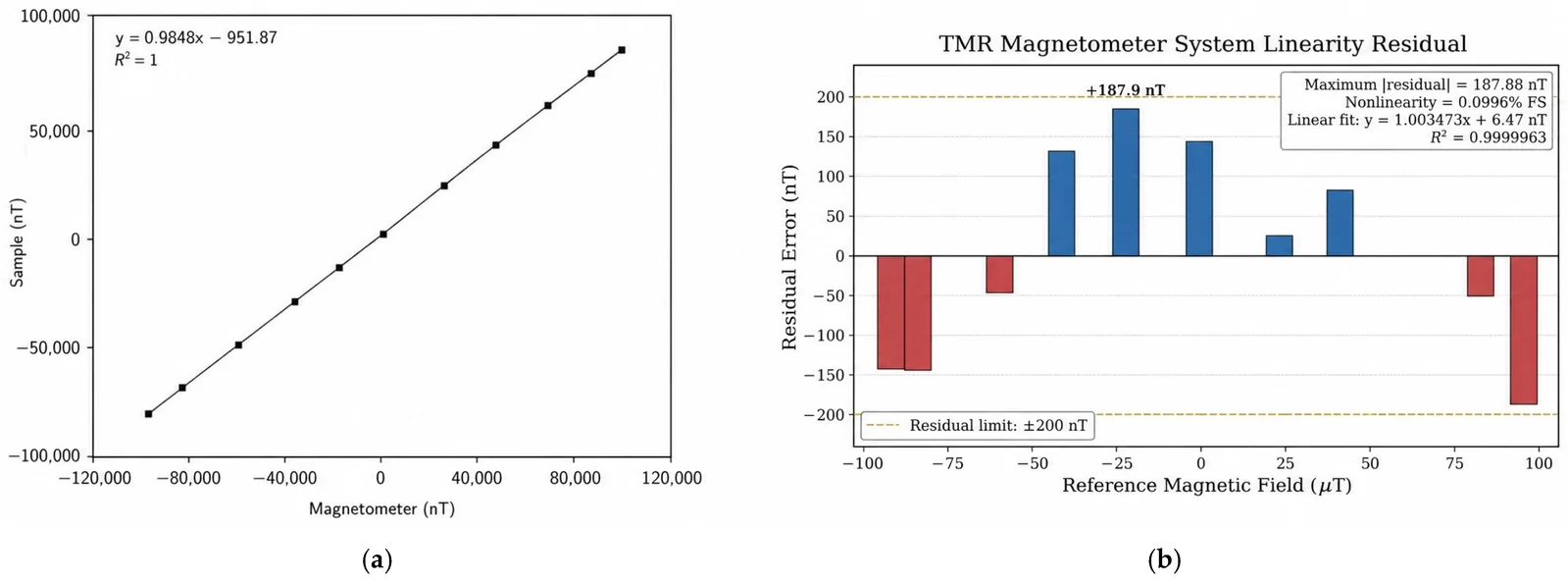
(**a**) Measured linearity and linear fitting results of the proposed TMR magnetometer. (**b**) The error in the measured linearity.

Figure 13 shows the linear fitting result between the output of the proposed TMR magnetometer and the reference magnetic field. The measured data exhibit excellent linearity over the entire ±100 μT measurement range. The coefficient of determination reaches R^2^ = 1, indicating an excellent linear relationship between the measured output and the applied magnetic field. Based on the maximum deviation from the ideal fitted line, the full-scale nonlinearity of the proposed system is 0.1% FS, demonstrating that the proposed interface ASIC is capable of accurately converting the weak differential output of the TMR sensor into highly linear digital magnetic-field measurements. The excellent linearity can be attributed to the proposed chopper-stabilized programmable instrumentation amplifier together with the digitally assisted offset calibration technique, which effectively suppresses offset errors and improves gain accuracy over the entire measurement range.

**C.**  **Noise Performance**

The noise performance of the proposed TMR magnetometer was evaluated under a near-zero magnetic-field environment. As discussed in the previous section, the input-referred noise of the instrumentation amplifier (IA) was characterized separately at the chip level. To ensure that the readout circuit does not degrade the overall magnetic-field resolution, the equivalent input noise of the interface ASIC should remain lower than the intrinsic noise floor of the TMR sensing element.

During the system-level noise measurement, the excitation current applied to the Helmholtz coils was turned off, and the TMR magnetometer prototype was positioned at the geometric center of the three-layer magnetic shielding chamber, where the residual magnetic field was approximately 1 nT. Under this condition, the sensor operated in an approximately zero-field environment, thereby minimizing the influence of external magnetic interference. The real-time digital output of the proposed interface ASIC was continuously acquired through the RS-232 interface by a PC. The sampling frequency was set to 30 Hz, and more than 5000 consecutive samples were collected for each measurement. The recorded data were subsequently processed using a fast Fourier transform (FFT) to obtain the magnetic noise power spectral density (PSD). To minimize the influence of the measurement setup and external excitation source on the low-frequency noise characterization, all measurements were performed inside a three-layer magnetic shielding chamber. During the noise measurements, the Helmholtz-coil current source was switched off and physically disconnected from the test system to eliminate excitation-related electrical coupling. Prior to characterizing the complete TMR sensor and ASIC system, the baseline noise of the data-acquisition chain was verified by short-circuiting its input under the same sampling and FFT-processing conditions. This procedure enabled the noise contribution of the measurement instruments to be distinguished from that of the sensor and readout circuit, thereby ensuring that the reported noise spectrum primarily reflects the intrinsic noise performance of the proposed TMR sensing system.

**Figure 14:**
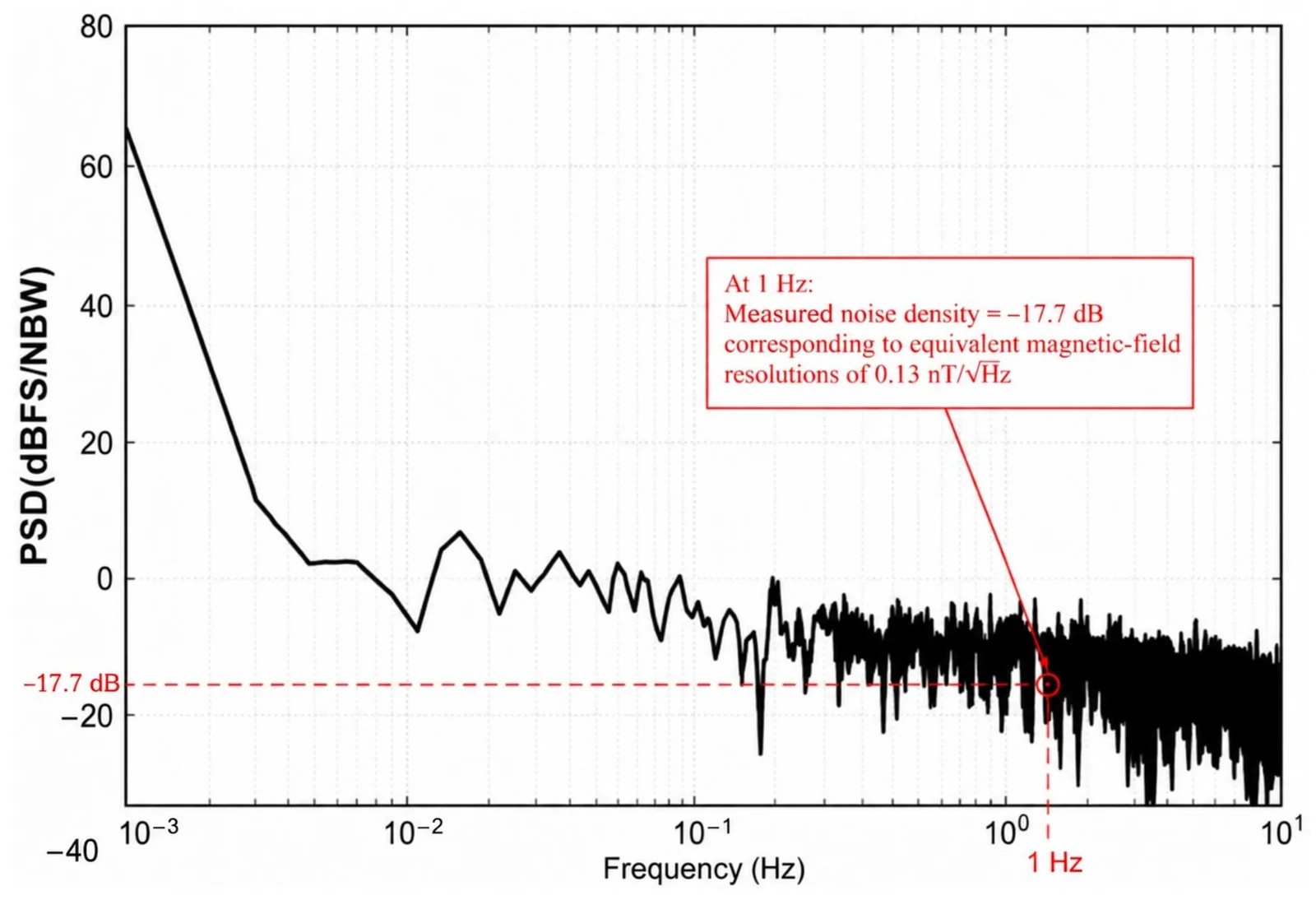
Measured magnetic noise power spectral density (PSD) of the proposed TMR magnetometer.

The measured noise spectra are shown in Figure 14. In the PSD plots, 0 dB corresponds to an equivalent magnetic noise density of 1 nT/√Hz, while 20 dB and −20 dB correspond to 10 nT/√Hz and 0.1 nT/√Hz, respectively. The measurement bandwidth covers the frequency range from 0.01 Hz to 10 Hz, which is suitable for evaluating the low-frequency performance of the proposed TMR magnetometer. At 1 Hz, the measured noise density is −17.7 dB, corresponding to an equivalent magnetic-field resolution of 0.13 nT/√Hz; the proposed readout circuit preserves the high magnetic-field resolution of the sensor while providing stable and low-noise signal conditioning for precision magnetic-field measurement. The excellent noise performance is mainly attributed to the proposed chopper-stabilized programmable instrumentation amplifier, the transconductance equalization technique, and the digitally assisted offset calibration circuit, which jointly suppress low-frequency flicker noise, minimize input offset, and improve the overall signal-to-noise ratio of the TMR sensing system.

Although numerous studies have investigated TMR sensing devices and magnetic readout circuits, reports of complete TMR-based systems specifically intended for ambient magnetic-field measurement remain comparatively limited. Many published studies focus on material-level or device-level characteristics and do not provide a complete set of system-level performance metrics. Moreover, the reported magnetic-noise density and resolution strongly depend on the sensor configuration, bridge-bias conditions, measurement bandwidth, and noise-evaluation frequency, making direct comparisons between different studies difficult. Therefore, the works included in Table 2 were selected based on their relevance to ambient magnetic-field sensing and the availability of sufficiently complete and comparable performance data. The comparison consequently emphasizes system-level consistency and technical comparability rather than simply increasing the number of references. Table 3 summarizes the performance comparison between the proposed magnetometer and representative state-of-the-art designs. Compared with existing implementations, the proposed system exhibits the lowest power consumption (10 mW) while maintaining excellent linearity (0.1% FS) and achieving the lowest magnetic noise density (0.13 nT/√Hz) among all reported 5 V designs. Although commercial magnetometers such as the HMR2300 provide acceptable linearity, they require substantially higher power consumption (400 mW) and exhibit much higher magnetic noise. Benefiting from the proposed chopper-stabilized programmable instrumentation amplifier together with the transconductance equalization and digitally assisted offset calibration techniques, the proposed system simultaneously achieves high sensitivity, low noise, and ultra-low power operation. Therefore, it provides a competitive solution for compact and high-resolution magnetic sensing systems.

## 6. Conclusions

This paper presents a low-power readout interface ASIC for TMR magnetic sensors based on a chopper-stabilized programmable instrumentation amplifier (PGIA). To improve the overall sensing performance, a transconductance equalization technique is proposed to maintain nearly constant input transconductance over the entire rail-to-rail common-mode range, thereby enhancing gain consistency and reducing input-referred noise. A dynamic slew-rate enhancement circuit is further introduced to improve the transient response without increasing the quiescent power consumption. In addition, a digitally assisted offset calibration circuit effectively suppresses the input offset, resulting in improved measurement accuracy and long-term stability. The proposed PGIA provides eight programmable gain settings from 1 V/V to 128 V/V, making it suitable for TMR sensors with different output amplitudes.

The proposed interface ASIC was fabricated using a 0.18 μm CMOS process and experimentally evaluated using a compact TMR magnetometer prototype. Measurement results demonstrate excellent linearity with a full-scale nonlinearity of 0.1% FS over a ±100 μT magnetic-field range. The measured magnetic noise density reaches 0.13 nT/√Hz at 1 Hz, while the total power consumption is only 10 mW under a 5 V supply. Compared with representative commercial and reported magnetometers, the proposed system achieves a favorable trade-off among power consumption, linearity, and magnetic-field resolution. These results verify the effectiveness of the proposed circuit techniques and demonstrate that the developed interface ASIC is well suited for portable, battery-powered, and high-resolution magnetic sensing applications.

Although the measured power consumption is lower than that of several previously reported high-resolution magnetometer systems, further reductions in supply voltage and standby current are required for severely energy-constrained or aggressively duty-cycled IoT nodes. Future work will focus on further reducing the system power consumption through supply-voltage optimization and integrating digital temperature compensation and self-calibration functions to improve long-term stability and environmental adaptability for practical applications.

## Figures and Tables

**Table 1:** Truth table of a 3-to-8 decoder.

A3	A2	A1	D8	D7	D6	D5	D4	D3	D2	D1
0	0	0	1	1	1	1	1	1	1	0
0	0	1	1	1	1	1	1	1	0	1
0	1	0	1	1	1	1	1	0	1	1
0	1	1	1	1	1	1	0	1	1	1
1	0	0	1	1	1	0	1	1	1	1
1	0	1	1	1	0	1	1	1	1	1
1	1	0	1	0	1	1	1	1	1	1
1	1	1	0	1	1	1	1	1	1	1

**Table 2:** Comparison of bias-current control techniques for rail-to-rail input stages.

Method	Bias-Current Control Principle	Additional Circuitry	*g_m_* Characteristics	Main Features
Conventional complementary input stage	Fixed tail currents for the NMOS and PMOS differential pairs	None	Varies by more than a factor of two	Simple implementation, but the GBW, input-referred noise, and gain vary with the input common-mode voltage
Three-times current-mirror method [44]	Increases the tail current of the remaining active pair from I to 4I	Current switches and 3:1 current mirrors	Approximately 15% variation in the takeover regions	Straightforward operating principle, but the high-ratio current mirrors increase circuit area and sensitivity to mismatch
Transition-region overlap method [45]	Uses DC level shifters to overlap the transition regions of the NMOS and PMOS differential pairs	DC level shifters and additional bias circuitry	Approximately ±4% measured variation	Provides smooth current handover, but requires relatively complex circuitry and accurate alignment of the transition regions
This work	Redistributes the bias current according to the conduction states of the complementary input pairs	Two switching transistors and asymmetric current mirrors	Approximately 211–224 μS, corresponding to a peak-to-peak variation of approximately 6%	Requires no separate common-mode detector or DC level shifter and is readily integrated with the chopper-stabilized folded-cascode PGIA

**Table 3:** Power-consumption comparison between the adaptive and fixed-output-stage amplifiers.

Miniaturized Magnetometer	Power/Range (V/Gauss)	Consumption (mW)	Nonliearity (%FS)	Noise Level (nT/√Hz)
HMR2300	12 V/±1–2 G	400	0.1–0.5	6.67
[46]	5 V/±1 G	77	0.2	0.15
[47]	5 V/±1 G	120	0.11	0.25
This work	5 V/±1 G	10	0.1	0.13

## Data Availability

The original contributions presented in this study are included in the article. Further inquiries can be directed to the corresponding authors.
